# Superovulation induces defective methylation in line-1 retrotransposon elements in blastocyst

**DOI:** 10.1186/1477-7827-11-69

**Published:** 2013-07-18

**Authors:** Xing-Wei Liang, Xiang-Shun Cui, Shao-Chen Sun, Yong-Xun Jin, Young Tae Heo, Suk Namgoong, Nam-Hyung Kim

**Affiliations:** 1 Department of Animal Sciences, Chungbuk National University, Cheongju 361-763, South Korea; 2 College of Animal Science and Technology, Nanjing Agricultural University, Nanjing 210095, China

**Keywords:** Superovulation, Oocyte, Pre-implantation development, DNA methylation, Dnmt

## Abstract

**Background:**

Series of epigenetic events happen during preimplantation development. Therefore assistant reproduction techniques (ART) have the potential to disrupt epigenetic regulation during embryo development. The purpose of this study was to investigate whether defects in methylation patterns in blastocyst due to superovulation originate from abnormal expression of Dnmts.

**Methods:**

Low- (6 IU) and high- (10 IU) dosage of PMSG was used to stimulate the female mice. The metaphase II(MII) oocytes, zygotes and blastocyst stage embryos were collected. Global methylation and methylation at H3K9 in zygote, and methylation at repeated sequence Line 1 and IAP in blastocysts were assayed. In addition, expression of Dnmts was examined in oocytes and zygotes.

**Results:**

Global DNA methylation and methylation at H3K9 in zygotes derived from females after low- or high-dosage hormone treatment were unaltered compared to that in controls. Moreover, DNA methylation at IAP in blastocysts was also unaffected, regardless of hormone dosage. In contrast, methylation at Line1 decreased when high-dose hormone was administered. Unexpectedly, expression of Dnmt3a, Dnmt3b, Dnmt3L as well as maintenance Dnmt1o in oocytes and zygotes was not disrupted.

**Conclusions:**

The results suggest that defects in embryonic methylation patterns do not originate from the disruption of Dnmt expression.

## Background

The use of ART (assisted reproductive techniques) for the treatment of human infertility/subfertility is rapidly increasing; according to estimates, in developed countries 1-2% of children are born via ART [1,2]. However, since the timing of ART coincides with that of key epigenetic events, epigenetic regulation may be susceptible to disruption. In particular, ovarian stimulation and *in vitro* culture have a high risk of DNA methylation disruption [3].

DNA methylation, the most characterized epigenetic modification, is involved in transcriptional repression, global X chromosome inactivation and genomic imprinting [4]. DNA methylation is catalyzed by a family of DNA methyltransferases (DNMTs). In mammals, five DNMTs (DNMT1, DNMT2, DNMT3A, DNMT3A and DNMT3L) have been defined [5]. They have two types of methyltransferase activity: *de novo* and maintenance methylation. DNMT3A and DNMT3B, the *de novo* DNMTs, play important roles in the establishment of DNA methylation patterns, but are dispensable for the maintenance of the methylation marks at most imprinted loci during preimplantation development [6]. DNMT1 (DNMT1s and DNMT1o being the somatic and oocyte-specific isoforms of DNMT1, respectively), a major maintenance DNMT, is essential for methylation maintenance. In mice, Dnmt1 alone is sufficient for the maintenance of methylation marks of imprinted genes during preimplantation development [6].

Methylation patterns required for genomic imprinting change dynamically during mammalian development. In general, imprinting is thought to be a multi-step process involving erasure, establishment and maintenance of a methylation mark. In the mouse, the paternal genome undergoes active global demethylation within 6–8 h of fertilization while passive demethylation of the maternal genome occurs during the cleavage stage [7,8]; however, imprinted genes and some repeated sequences maintain methylation during this wave of global demethylation [9]. Imprints are erased in primordial germ cells and must then be re-established during gametogenesis in a sex-specific manner [10,11]. During oogenesis, maternal imprints are acquired at a specific time for each gene during postnatal oocyte growth. This process was also found to correlate with an increase in oocyte diameter [12,13]. During spermatogenesis, paternal imprints are established in the postnatal stage [14,15]. If methylated imprints are incorrectly established during gametogenesis, these defects may be found in the resulting pregnancy.

Studies have been carried out to determine the effect of ovarian stimulation on DNA methylation during oogenesis, preimplantation and postimplantation development [3]. Interestingly, the results reported are conflicting. For oocytes, Sato et al. [16] demonstrated a gain in *H19* methylation in mouse oocytes and in human oocytes derived from superovulated females, but a loss of *PEG1* methylation in the latter. In contrast, Anckaert et al. [17] found that superovulation did not affect methylated imprint acquisition at *H19*, *Snrpn* or *Igf2r* in mouse oocytes. Moreover, a recent study showed that *H19*, *Snrpn*, *Peg3* and *Kcnq1ot1* have normal methylated imprint patterns in mouse oocytes when low- or high-dose hormone was administered [18]. During preimplantation development, *H19* methylation was unaltered, whereas in mouse blastocysts *H19* expression was disturbed [19]; however, loss of methylation at the maternally imprinted genes *Snrpn*, *Peg3* and *Kcnq1ot1* and gain of methylation at the paternally imprinted gene *H19 *were observed in blastocysts by Market-Velker et al. [20]. Similarly, global methylation in 2-cell stage embryos derived from superovulated mice was two times higher than that in non-superovulated counterparts [21]. During postimplantation development, superovulation resulted in biallelic expression of *Snrpn* and *H19 *at 9.5dpc (day post coitus) placentas while *Kcnq1ot1*expression was not affected [22]. Interestingly, DNA methylation at *Snrpn* and *H19* was unaltered [22]. Taken together, these data show that the effect of superovulation on DNA methylation varies and is incompletely understood.

Ovarian stimulation was found to disturb DNA methylation at not only maternally but also paternally imprinted genes in mouse blastocysts [20]. Such dual effects on methylated imprints may originate from abnormal imprint acquisition or from disruption of maternal-effect gene production, which is required for later imprint maintenance. Abnormal methylated imprint acquisition cannot explain methylation defects in blastocysts since methylated imprints are correctly established in oocytes [18]. Therefore, defects in imprint maintenance in blastocyst are suspected to originate from the disruption of gene products stored in oocytes.

Long interspersed elements-1 (LINE-1) is highly repeated human retrotransposon elements found in large numbers in eukaryotic genomes [23]. LINE-1s constitute about 17% of human genome as 600,000 copies and about 3000–400 copies of LINE-1s remains as full length form and some of them may retain its activity [24]. The intracisternal A-particle (IAP) element is a long terminal repeat (LTR)-type mouse retrotransposon, which is consisted with gag, pro, and pol genes [25]. Recently it was previously reported that the most extreme methylation changes during the sperm to zygote transition are enriched for LINEs [26]. Especially LINE L1 is the one with most significant decrease in methylation level, while class II intercisternal A-particles (IAP) does not show any methylation within zygotes. LINE and LTR activity in the early embryo is associated with some of the earliest transcriptional events during zygotic genome activation [27].

The objective of the present study was to further investigate the effect of superovulation on methylation and to determine whether the expression of Dnmts, which is required for methylation acquisition and maintenance, is disturbed upon ovarian stimulation. Two hormone dosages, low (6 IU) or high (10 IU), were administered. Dnmt mRNA levels were assessed in metaphase II (MII) oocytes and zygotes. In addition, to further understand the effect of superovulation on methylation, global DNA methylation and H3K9 methylation were investigated in zygotes, and DNA methylation was assessed at repeated sequence (IAP LTR and Line1 5′)in blastocyst stage embryos.

## Methods

### Ovarian stimulation, oocyte, zygote and blastocyst collection

ICR strain female mice 8–12 weeks of age and male mice 12–24 weeks of age were used in this study. The mice were sacrificed by cervical dislocation. All animal manipulations were conducted according to the guidelines of the Animal Research Committee of Chungbuk National University.

For ovarian stimulation, females were administered a single dose of PMSG (pregnant mare serum gonadotropin) followed by the same dosage of hCG (human chorionic gonadotropin) after 48 h. Hormone doses of 6 IU (low dose) and 10 IU (high dose) were administered. To obtain MII oocytes, mice were sacrificed and oocyte-cumulus cell complexes were collected into M2 medium (Sigma) 15 h after hCG injection. Oocyte-cumulus cell complexes collected from untreated females in the estrous stage of spontaneous ovulation cycles were used as controls. Cumulus cells were removed with 0.03% hyaluronidase in M2 medium. Oocytes were washed extensively with M2 medium.

To obtain pronuclear-stage zygotes and blastocyst-stage embryos from ovarian stimulation, females were administered hCG and were then mated with males in the afternoon. The presence of a vaginal plug was assessed in the morning following mating. Zygotes were collected from the oviduct of vaginal plug-positive females into M2 medium 22–24 h after hCG injection. The cumulus cells surrounding the zygotes were removed with 0.03% hyaluronidase in M2 medium and the zygotes were extensively washed with M2 medium. Blastocysts were flushed from the uterus of vaginal plug-positive females ~96 h after hCG injection. Control zygotes and embryos at the same stage as those of the hormone treatment groups were collected from spontaneously ovulating females mated with males. The oocytes, zygotes and blastocysts collected were stored at −80°C or fixed for later use.

### Immunostaining and confocal microscopy

Zygotes were washed several times in PBS (phosphate-buffered saline), fixed for 20 min in 3.7% paraformaldehyde in PBS, and permeabilized with 0.5% Triton X-100 at room temperature for 20 min. Thereafter, the permeabilized zygotes were blocked in 1% BSA (bovine serum albumin) in PBS (blocking buffer) for 1 h at room temperature, and then incubated in anti-H3K9dim antibody (1:300) (Cell Signaling) in blocking solution overnight at 4°C. After overnight incubation, the zygotes were washed several times in washing buffer (0.1% Tween and 0.01% Triton X-100 in PBS), transferred to Alexa Fluor 488 goat anti-rabbit (1:200) (Invitrogen), and incubated for 1 h at room temperature. The zygotes were co-stained with Hoechst 33342 (10 ug/ml) for 15 min and washed three times in washing buffer. The samples were mounted on glass slides and evaluated with a confocal laser-scanning microscope (Zeiss LSM 710 META).

The procedure for detection of DNA methylation is similar to that of H3K9 with the following exceptions. After washing several times in PBS, the zygotes were fixed in 3.7% paraformaldehyde and permeabilized with 0.5% Triton X-100. Subsequently, the permeabilized zygotes were incubated in 4 N HCl solution at room temperature for 10 min followed by neutralization in Tris-Cl solution (pH 8.0) for 10 min. After blocking, the zygotes were incubated in 5mC antibody (1:300) (Calbiochem) overnight at 4°C. The next steps were similar to those for H3K9 detection, with the exception that the zygotes were co-stained with Alexa Fluor 488 goat anti-mouse (1:200) (Invitrogen) for 1 h and PI (10 ug/ml) at 37°C for 30 min.

### mRNA extraction and real-time PCR

Poly(A) mRNA was extracted from at least 50 MII oocytes or zygotes using the Dynabeads mRNA Direct Micro kit (Invitrogen). Oligo (dt) and extracted poly (A) mRNA were used to prepare cDNA using Superscript III reverse transcriptase (Invitrogen). All procedures were performed according to the manufacturer’s instructions.

For PCR, primer and cDNA template were added to SYBR Green Real-Time PCR Master Mix (Toyobo). PCR consisted of one cycle at 95°C for 2 min, 40 cycles at 95°C for 15 s, 60°C for 15 s and 72°C for 45 sin a CFX real-time PCR cycler (Bio-Rad). The cycle threshold (Ct) values used for calculating relative expression were the averages of three replicates and were normalized to those of two reference genes (Gapdh and Atp5b). Dissociation curves were used to assess the specificity of the PCR products. Expression levels were calculated using the 2^-△△Ct^ method as described previously [28,29]. The primers used for qRT-PCR are listed in Table 1. At least three replicates were performed for each experiment. Statistical analyses were conducted using an analysis of variance. Differences between treated groups were evaluated with Duncan’s multiple comparison tests. Data were expressed as mean + SEM, and P < 0.05 was considered significant.

**Table 1:** **qRT**-**PCR and BS primers**

	**Gene**	**Primers**	**Ref.**
*qRT*-*PCR*	Dnmt1o	F: 5′-GGTTGATTGAGGGTCATT-3′	[34]
		R: 5′-GCAGGAATTCATGCAGTAAG-3′	
	Dnmt3a	F: 5′-GCCGAATTGTGTCTTGGTGGATGACA-3′	[36]
		R: 5′-CCTGGTGGAATGCACTGCAGAAGGA-3′	
	Dnmt3b	F: 5′-TTCAGTGACCAGTCCTCAGACACGAA-3′	[36]
		R: 5′-TCAGAAGGCTGGAGACCTCCCTCTT-3′	
	Dnmt3L	F: 5′-GTGCGGGTACTGAGCCTTTTTAGA-3′	[36]
		R: 5′-CGACATTTGTGACATCTTCCACGTA-3′	
	Atp5b	F: 5′-GGCCAAGATGTCCTGCTGTT-3′	[30]
		R: 5′-GCTGGTAGCCTACAGCAGAAGG-3′	
	Gapdh	F: 5′-GCCGGGGCTGGCATTGCT-3′	
		R: 5′-TTGCTCAGTGTCCTTGCTGGGG-3′	
*BS*	IAP LRT	F1: 5′-TTGATAGTTGTGTTTTAAGTGGTAAATAAA-3′	[9]
		R1: 5′-AAAACACCACAAACCAAAATCTTCTAC-3′	
		F2: 5′-TTGTGTTTTAAGTGGTAAATAAATAATTTG-3′	
		R2: 5′-CAAAAAAAACACACAAACCAAAAT-3′	
	Line1 5′	F1: 5′-GTTAGAGAATTTGATAGTTTTTGGAATAGG-3′	[9]
		R1: 5′-CCAAAACAAAACCTTTCTCAAACACTATAT-3′	
		F2: 5′-TAGGAAATTAGTTTGAATAGGTGAGAGGT-3′	
		R2: 5′-TCAAACACTATATTACTTTAACAATTCCCA-3′	

### Bisulfite modification and sequencing

Three of each set often blastocysts obtained from multiple control or hormone-treated females were subjected to modification using the EZ Methylation Direct kit (Zymo Research), according to the manufacturer’s instructions. The methylation of partial regions at Line1 5′ and IAP LTR, which respectively contain 9 and 11 CpG sites, was assessed. Nested-PCR was performed to amplify bisulfite-modified DNA. Primer sequences are shown in Table 1. The PCR settings previously reported by Lane et al. were used [9]. PCR products were run in 1.5% agarose gel. Specific products were extracted using the QIAEX II gel extraction kit (Qiagen) and then ligated into the pGEM-T easy vector (Promega). Plasmids were extracted from positive clones using the Quick Lyse Miniprep kit (Qiagen) and sequenced using the M13 forward or M13 reverse sequencing primer.

## Results

### Global DNA methylation and H3K9 methylation profiles in zygotes

**Figure 1:**
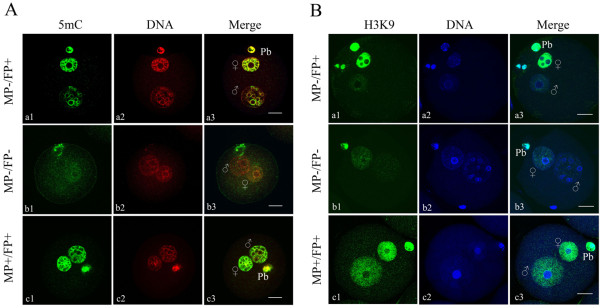
**Distribution patterns of global DNA methylation (A) and methylation at histone H3K9 (B) in pronucleus**-**stage mouse zygotes.** The zygotes were stained with 5mC-specific antibody (green) and counterstained with DAPI (red), or stained for H3K9 dimethylation (green) and counterstained with Hoechst 33342 (blue). MP-/FP + indicates absence of a signal in male pronuclei and presence of a signal in female pronuclei; MP-/FP- indicates that both pronuclei had a very weak signal or lacked a signal; and MP+/FP + indicates both pronuclei had a signal. The polar body (Pb) was always positively stained. MP: male pronucleus; FP: female pronucleus; (+) positive signal; (−) negative or weak signal.

Methylation profiles were assessed in zygotes derived from control and superovulated females using specific antibodies to 5mC and H3K9. As shown in Figure 1, three types of signals were observed in paternal and maternal pronuclei. Zygotes in which the female pronuclei were positive while the male pronuclei were negative or displayed only weak signals were designated FP+/MP-. Zygotes in which both the female and male pronuclei were negative or displayed only weak signals were designated FP-/MP-. Zygotes in which both the female and male pronuclei were positive were designated FP+/MP+. The polar body was always strongly stained and used to assess antibody accessibility and staining quality.

5mC staining results showed that most of the control zygotes (89%) displayed the FP+/MP- pattern. Most zygotes derived from hormone-treated females also showed the FP+/MP- pattern; 94% and 89% of the low- and high-dose hormone treatment groups showed FP+/MP- pattern, respectively (Table 2). Compared to control zygotes, no significant change in global methylation was found in zygotes derived from hormone-treated females, regardless of hormone dosage. As with 5mC staining, H3K9 staining showed that greater than 90% of the zygotes in each group displayed the FP+/MP- pattern; the percentages of zygotes displaying the FP+/MP- pattern were 94%, 93% and 92% for control, low- and high-dose hormone-treated females, respectively (Table 2). The analysis showed no significant difference between the three groups. The results of 5mC and H3K9 staining clearly demonstrated that approximately 90% of the zygotes were demethylated in male pronuclei, while almost all of the zygotes maintained methylation in female pronuclei regardless of ovarian stimulation, suggesting that ovarian stimulation did not affect global methylation levels in zygotes. In the present study, at least 80 zygotes derived from three separate batches of zygotes were examined for each group.

**Table 2:** DNA methylation profiles in zygotes derived from control and superovulated females

		**5mC**	**H3K9**
*Control*	MP-/FP+	76 (89%)	63 (94%)
	MP-/FP-	4	0
	MP+/FP+	5	4
	Total	85	67
*Low dose*	MP-/FP+	107 (94%)	108 (93%)
	MP-/FP-	0	2
	MP+/FP+	7	6
	Total	114	116
*High dose*	MP-/FP+	104 (89%)	103 (92%)
	MP-/FP-	5	6
	MP+/FP+	8	3
	Total	117	112

### IAP and Line1 DNA methylation profiles in blastocysts

**Figure 2:**
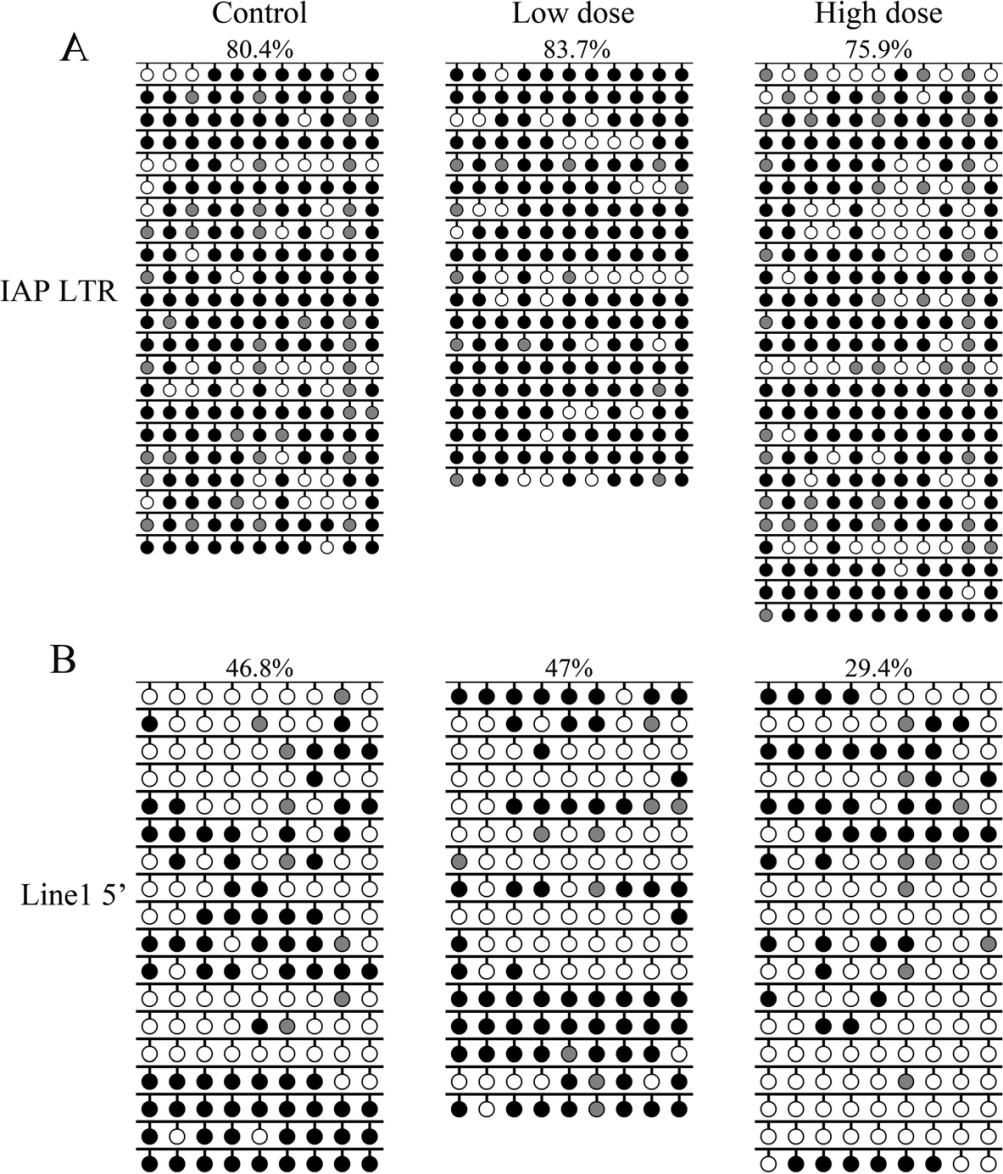
**IAP and Line1 DNA methylation profiles in blastocysts.** Sodium bisulfite sequencing was used to examine the DNA methylation patterns of **(A)** IAP LTR and **(B)** Line1 5′ end sequences in blastocyst DNA. Blastocysts were obtained from control, low- and high-dose hormone treatment groups. Target sequences were amplified, cloned and sequenced. Open circles, unmethylated CpGs; black circles, methylated CpGs; gray circles, not analyzable/mutated CpG site. Each row represents an individual sequenced clone. Only black and white circles were analyzed. The percentage of methylated CpGs (black circles/(black + white circles)) is indicated.

Although the effect of superovulation on DNA methylation patterns at imprinted sequences has previously been investigated in mouse blastocysts, the effect of superovulation on the DNA methylation of repeated sequences has not yet been assessed. To examine whether superovulation influences the methylation pattern of repeated sequences, the methylation levels of IAP and Line1 were assayed in blastocyst-stage embryos. Sequencing results showed that IAP methylation levels were similar in blastocysts derived from spontaneously ovulating females (80.4%) and superovulated females treated with low-dose (83.7%) or high-dose (75.9%) hormone (Figure 2). Line1 methylation levels were 46.8%, 47% and 29.4% for the spontaneously ovulating, low- and high-dose superovulation groups, respectively (Figure 2). The Line1 methylation level in the low-dose group was close to that of the control group, whereas that of the high-dose group was about 17% lower than the controls. Our data showed that superovulation with lose-dose hormone did not affect the methylation levels of the repeated sequences IAP and Line1; however, the Line1 methylation level was decreased in superovulated females treated with high-dose hormone while the methylation of IAP was unaltered.

### Expression of Dnmts in MII oocytes and zygotes

**Figure 3:**
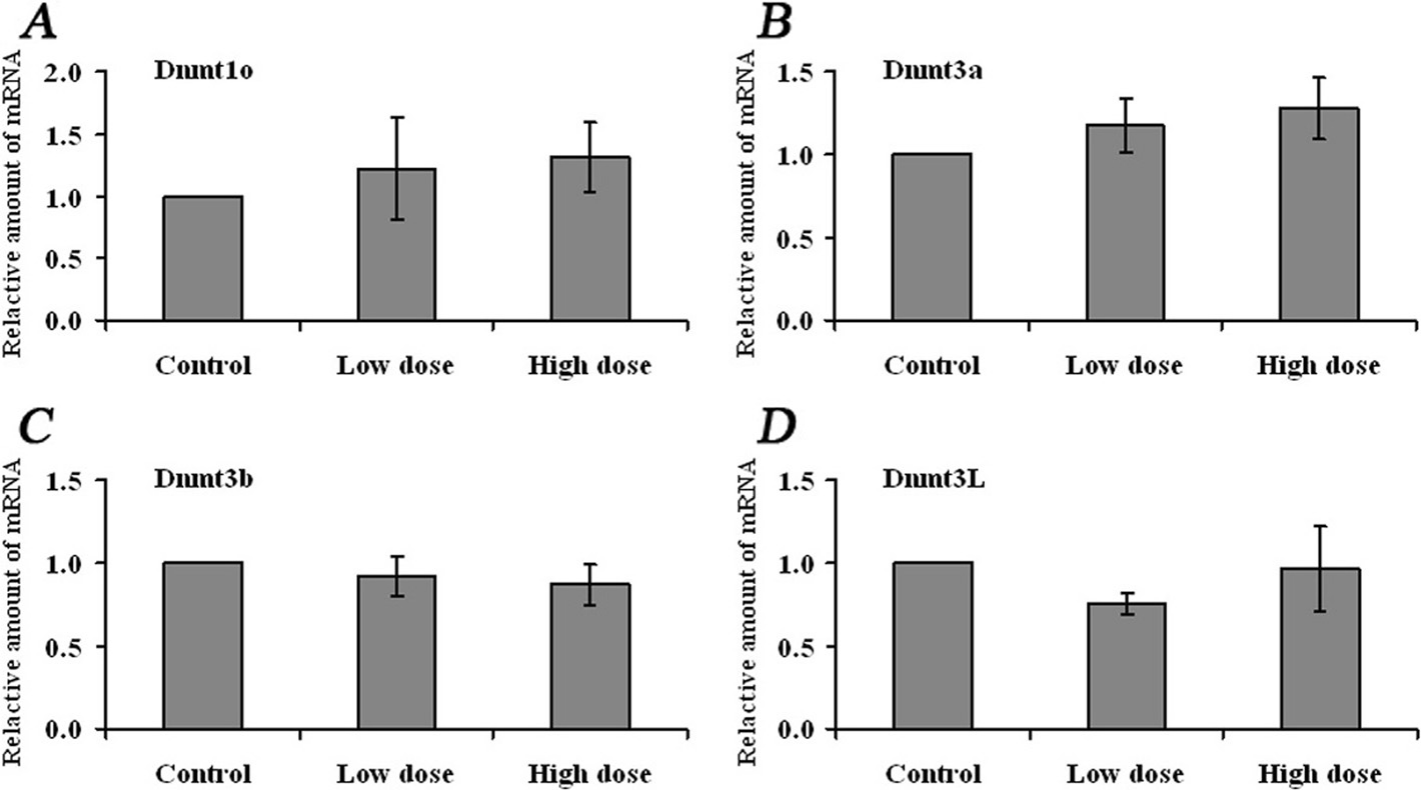
**Expression of Dnmts in MII oocytes derived from control and superovulated females.** qRT-PCR was applied to evaluate the mRNA abundance of **A)** Dnmt1o, **B)** Dnmt3a, **C)** Dnmt3b and **D)** Dnmt3L in MII oocytes derived from control, low- (6 IU) and high-dose hormone treatment groups, respectively. Two reference genes, Gapdh and Atp5, were used for each sample. Data were normalized to the average levels of the two reference genes. The fold change was calculated using the comparative C_T_ method. Data are presented as mean ± SD.

To determine whether oocyte Dnmt transcripts were affected by superovulation, Dnmt3a, Dnmt3b, Dnmt3L and Dnmt1o were assayed by quantitative real-time PCR. MII oocytes were collected after spontaneous cycle and ovarian stimulation. The results showed that the transcription of these four genes was not altered by superovulation regardless of hormone dosage (Figure 3).

**Figure 4:**
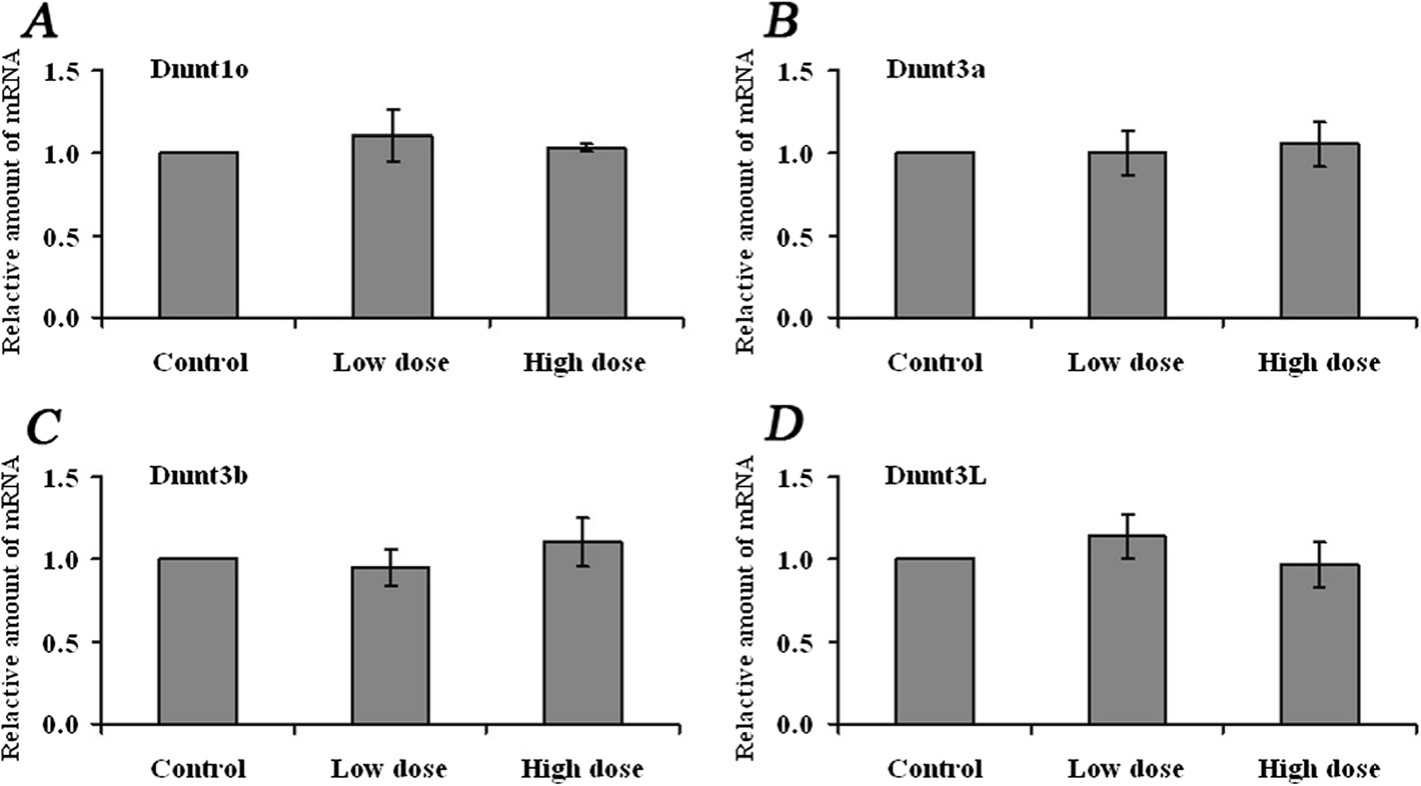
**Expression of Dnmts in zygotes derived from control and superovulated females.** qRT-PCR was applied to evaluate the mRNA abundance of **A)** Dnmt1o, **B)** Dnmt3a, **C)** Dnmt3b and **D)** Dnmt3L in zygotes derived from control, low- (6 IU) and high-dose hormone treatment groups, respectively. Data were normalized to the average levels of Gapdh and Atp5 for each sample. The fold change was calculated using the comparative C_T_ method. Data are presented as mean ± SD.

The transcript levels of Dnmt3a, Dnmt3b, Dnmt3L and Dnmt1o were further examined in pronuclear zygotes. The zygotes used for transcript analysis and those used for methylation analysis were derived from the same sources. The mRNA levels of each gene were compared in zygotes from the control, low- and high-dose hormone groups. As in oocytes, Dnmt mRNA levels in zygotes were unaffected (Figure 4). These results indicate that superovulation did not disrupt Dnmt transcript levels in oocytes or zygotes.

## Discussion

Although studies in recent years have suggested that defects in imprint maintenance in blastocysts due to superovulation may originate from the disruption of maternal-effect gene products that are required for imprint maintenance after fertilization, this mechanism has yet to be confirmed. In the present study, the expression of Dnmts was examined in oocytes and zygotes obtained from superovulated females. In addition, global DNA methylation and H3K9 methylation levels were examined in zygotes, and IAP and Line1 methylation was assessed in blastocysts. Since previous studies on the effect of superovulation on DNA methylation yielded conflicting results, a superovulation regimen that was shown to affect methylation maintenance in blastocysts, rather than methylation acquisition in oocytes, was chosen [18,20]. The results demonstrated that only DNA methylation at Line1 in blastocysts was adversely affected by high-dose hormone treatment; however, Dnmts were normally expressed in oocytes and zygotes obtained from females after hormone treatment.

### Superovulation did not affect global DNA methylation or methylation at H3K9

It is well established that the paternal genome undergoes active demethylation shortly after fertilization [7,8,30]. Immunostaining and confocal microscopy were used to determine whether this earliest epigenetic reprogramming event after fertilization was disrupted by superovulation. Similar to a previous study by Santos et al. who found that the male pronucleus was completely demethylated in mouse zygotes within several hours post fertilization [8], our results showed that the paternal genome undergoes active demethylation in control pronuclear zygotes and most female zygotes after low- and high-dose hormone stimulation. In contrast, Shi and Haaf found *in vivo* that 2-cell stage embryos from superovulated females showed 10% more defects in global methylation compared to counterparts from non-superovulated females [21]. Given the fact that superovulation did not affect global methylation in pronuclear zygotes in this study, and given previous data showing disrupted global methylation in 2-cell stage embryos, hormone treatment may only begin to affect global methylation patterns in embryos after the zygotic stage.

### Methylation of Line1 decreased after high-dose hormone treatment

The effect of superovulation on methylated imprints of single-copy sequences in blastocysts has been investigated by Market-Velker et al. [20] but extent of methylation on repeated elements during preimplamentation development have been obscured. Recently it was reported that the most extreme methylation changes during sperm to zygote transition are enriched for LINEs by genome wide bisulfide sequencing [26]. Especially LINE L1 is the one with most significant decrease in methylation level, while class II intercisternal A-particles (IAP) does not show any methylation changes in zygotes. In our studies, the DNA methylation profiles of two repeated sequences, IAP and Line1, were examined in blastocysts derived from spontaneously ovulating females and from females treated with low-or high-dose hormone. The results showed that Line1 undergoes substantial demethylation during preimplantation development, while IAP is largely resistant to demethylation during this period [9] IAP methylation levels were approximately similar in all groups. Whereas Line1 methylation patterns were unaffected by low-dose hormone treatment, Line1 methylation levels decreased when high-dose hormone was administered. These data indicate that methylation at Line1 is susceptible to disruption by ovarian stimulation. Previously, Merket-Velker et al. demonstrated that imprinting defects caused by ovarian stimulation were dose-dependent, with aberrant imprinted methylation being more frequent at high hormone doses [20]. Similarly, the current study showed that the disruption of methylation at repeated elements by ovarian stimulation is dose-dependent.

Why superovulation causes DNA demethylation in Line1 elements? In human, ovarian stimulation has been shown to accelerate the growth rate of ovarian follicles [31], this shortened maturation by superovulation may cause imcomplete acquisition of imprinting marks on the maternal alleles [20]. Lines and long tandem repeat (LTR) activity in the early embryo is associated with some of the earliest transcriptional events during zygotic genome activation and antisense oligonucleotide against Line1 caused a complete and irresponsible arrest of development [27]. These results suggest that importance of methylation status of Line1 during preimplantation development and superovulation may disturb the balance for methylation and demethylation on Line1 element. But the exact molecular mechanism for Line1 specific demethylation by superovulation remained to be elucidated.

### Normal expression of Dnmts in MII oocytes and zygotes

Methylation acquisition of imprinted genes and repeated sequences in oocytes during oogenesis is catalyzed by the *de novo* DNA methyltransferases Dnmt3a, Dnmt3b, Dnmt3L. Dnmtexpression profiles in postnatal oocytes during the period when methylation patterns are being established have been determined by Lucifero et al. [32]. We examined the effect of superovulation on the expression of Dnmtsin MII oocytes and zygotes, and found that Dnmt3a, Dnmt3b, Dnmt3L have normal expression compared to that of controls. Our results further confirmed the previous investigation by Denomme et al. who found that acquisition of methylation during oogenesis was unaffected by superovulation [18]. In addition, methylation patterns were also normally established in oocytes derived from follicles cultured *in vitro* and exposed to hormone [17]. Interestingly, Sato et al. demonstrated in superovulated females a gain in *H19* DNA methylation in mouse and human oocytes and a loss of *Peg1* methylation in human oocytes [16]. The altered methylation pattern at imprinted genes in oocytes observed by Sato et al. may be caused by multiple dose regimens or by infertility factors in humans [16].

Besides Dnmt3a, Dnmt3b, Dnmt3L the expression levels of Dnmt1o were evaluated in oocytes and zygotes. Dnmt1o is the oocyte-specific isoform of Dnmt1 and lacks the 118 amino acids of the N-terminal domain of the somatic isoform of Dnmt1 [33,34]. Dnmt1o was highly expressed in MII oocytes and in one-cell embryos [34]. Whereas genomic methylation patterns were normally established in Dnmt1o-deficient oocytes, embryos derived from such oocytes showed a loss of allele-specific expression and methylation at certain imprinted loci [35]. We expected that expression of Dnmt1o in oocytes and/or zygotes would be disturbed by hormone treatment since superovulation disrupts the DNA methylation maintenance of imprinted genes and repeated sequences in blastocysts. Unexpectedly, the expression of Dnmt1o in MII oocytes and zygotes was unaffected by superovulation. These results are not sufficient to explain the cause of the methylation defects at imprinted genes and repeated sequences observed in blastocysts induced by ovarian stimulation. Given the fact that normal expression of Dnmt1o and the defects in methylation maintenance in blastocysts, it is possible that superovulation induces Dnmt1o protein or enzyme activity decrease, subsequently lead to improperly maintained methylation patterns in imprinted genes and repeated sequences during preimplantation development.

In the current study, we investigated the effect of superovulation on global methylation in zygotes as well as on the methylation of repeated sequences in blastocysts. The results should help further understanding of the effects of superovulation on DNA methylation during preimplantation development. In addition, we evaluated the expression levels of *de novo* and maintenance methyltransferases in oocytes and zygotes derived from superovulated females, and found that Dnmts were normally expressed.

## Conclusions

Together with previous results showing that superovulation affects methylation maintenance during preimplantation rather than methylation acquisition during oogenesis [18,20], the present data showing unchanged Dnmt expression indicates that defects in methylation maintenance in blastocysts may not originate from disruption of Dnmt1o expression.

## Competing interests

The authors declare that they have no competing interests.

## Authors’ contribution

XWL and XSC carried out the experiments and wrote the first draft. SCS participated in the experimental design and statistics. YXJ helped in the experiments and did the assays. NHK conceived the idea and supervised the experiments. SN and YTH participated in writing and revising manuscripts. All authors read and approved the final manuscript.
